# Brain Shuttle Neprilysin reduces central Amyloid-β levels

**DOI:** 10.1371/journal.pone.0229850

**Published:** 2020-03-10

**Authors:** Christopher R. Campos, Alicia M. Kemble, Jens Niewoehner, Per-Ola Freskgård, Eduard Urich

**Affiliations:** 1 Neuroscience Discovery, Roche Pharma Research and Early Development, Roche Innovation Center Basel, Basel, Switzerland; 2 Large Molecule Research, Roche Pharma Research and Early Development, Roche Innovation Center Munich, Munich, Germany; Nathan S Kline Institute, UNITED STATES

## Abstract

Reducing Amyloid β (Aβ) in the brain is of fundamental importance for advancing the therapeutics for Alzheimer`s disease. The endogenous metallopeptidase neprilysin (NEP) has been identified as one of the key Aβ-degrading enzymes. Delivery of NEP to the brain by utilizing the Brain Shuttle (BS) transport system offers a promising approach for clearing central Aβ. We fused the extracellular catalytic domain of NEP to an active or inactive BS module. The two BS-NEP constructs were used to investigate the pharmacokinetic/pharmacodynamics relationships in the blood and the cerebrospinal fluid (CSF) in dose-response and multiple dosing. As previously shown, NEP was highly effective at degrading Aβ in blood but not in the CSF compartment after systemic administration. In contrast, the NEP with an active BS module led to a significant CSF exposure of BS-NEP, followed by substantial Aβ reduction in CSF and brain parenchyma. Our data show that a BS module against the transferrin receptor facilitates the transport of an Aβ degrading enzyme across the blood-brain barriers to efficiently reduce Aβ levels in both CSF and brain.

## Introduction

The incidence of Alzheimer`s disease (AD), the most common cause of dementia, rises dramatically with age[1]. The accumulation of neuro-toxic β-amyloid (Aβ) species in the brain is believed to contribute to the pathology of AD. Aβ is produced through the cleavage of the amyloid precursor protein to release primarily soluble Aβ monomers (Aβ_40_ and Aβ_42_)[2]. Aβ accumulation could either be due to increased production or decreased clearance of Aβ and therapies are being developed to tackle both mechanisms in AD patients[3]. The challenge to bring clinically successful therapeutics to the market for the treatment of AD has been an intensive but unfulfilled pursuit for almost three decades[4]. Enzymatic degradation of Aβ monomers has received attention over the past decade. Among the identified enzymes capable of degrading Aβ into less neuro-toxic fragments, Neprilysin (NEP) appears to have the greatest potential to effectively degrade toxic Aβ species[5]. Multiple lines of evidence highlight the crucial role of NEP in AD and indicate that increasing activity of NEP in the brain could have therapeutic potential in treating AD[6]. Although NEP is very effective in degrading Aβ in the blood when peripherally administrated, its access to the brain remains a key challenge[5]. Similar to other large molecules, NEP is unable to cross the blood-brain barrier (BBB) and requires brain delivery strategies. In this study we therefore sought to determine whether using transferrin receptor (TfR)-mediated transport at the BBB could induce the reduction of brain Aβ levels. TfR is present at relatively high levels on the brain vasculature and a TfR single chain Fab antibody (Brain Shuttle module) was recently used to efficiently deliver antibodies into the murine brain[7]. In our present study a Brain Shuttle (BS) module derived from a monoclonal antibody (OX26) selective to rat TfR[8] was fused to human NEP (hNEP) to facilitate the transport of the peripherally administered hNEP into the rat brain. To measure hNEP and Aβ in the cerebrospinal fluid (CSF) we conducted the studies in conscious cisterna magna cannulated naïve rats with normal physiological Aβ levels. Continuous CSF and blood sampling after peripheral administration of the hNEP with the inactive BS module led to sustained plasma Aβ reduction, but minimal CSF exposure and thus no impact on CSF and brain parenchyma Aβ levels. However, hNEP fused to an active BS module led to increased CSF and brain exposure with a reduction in Aβ levels in both CSF and brain parenchyma. This was shown both in a dose-dependent manner and administered on a multiple-dosing regimen.

## Materials and methods

### Generation of fusion proteins

NEP fusion proteins were generated by fusing the extracellular domain of human NEP (residues 52–750) to the C-terminus of an IgG1 heavy chain consisting of the mouse variable domain of the anti-rat TfR antibody OX26[9] and a human IgG1 constant region modified to include the “knob” mutation, paired with a human IgG1 Fc including the “hole” mutations. For the control construct, the OX26 variable region was replaced by that of a non-binding control antibody. Fusion proteins were expressed in transiently transfected HEK293 cells and purified via protein A affinity and size exclusion chromatography. Recombinant human NEP (control for biochemical activity) was obtained from R&D Systems.

### Determination lof enzymatic activity

The 20 μ assay was performed on low-volume black Costar 384-well plates at 25 C°. A working solution of 160 μM peptide substrate MCA-RPPGFSAFK(Dnp)-OH (R&D Systems) was prepared in 50 mM Tris-HCl pH7.8, 25 mM NaCl and 5 mM ZnCl2. 10 μl of NEP (R&D Systems) or NEP fusion polypeptide, diluted to 1, 2 or 4 nM in assay buffer, were transferred to plate. For determination of apparent *K*_*M*_ values various concentrations of substrate (0.078–80 nM in 2-fold dilutions) were added and the enzyme reaction started. The fluorescence increase was monitored with excitation at 320 nm and emission at 405 nm on an Envision Reader. Hydrolysis rates and apparent *K*_*M*_ values were calculated using XLFit^®^ software (IDBS).

### Rat TfR binding by flow cytometry

Binding of fusion polypeptide to rat transferrin receptor was tested by FACS analysis on rat C6 glioma cells. Cells were harvested by centrifugation, washed once with PBS and 5 x 10^4^ cells incubated with a 1.5 pM to 10 nM dilution series of the polypeptide fusions in 100 μL RPMI/10% FCS for 1.5 h on ice. After 2 washes with RPMI/10% FCS, cells were incubated with goat-anti-human IgG coupled to Phycoerythrin (Jackson Immunoresearch) at a dilution of 1:600 in RPMI/10% FCS for 1.5 h on ice. Cells were again washed, resuspended in RPMI/10% FCS and Phycoerythrin fluorescence measured on a FACS-Array instrument (Becton-Dickinson).

### Surgical implantation of the cannula in the cisterna magna

The cisterna magna (CM) of anesthetized male Wistar rats (200–350g) was permanently cannulated by using the methods previously described[9]. Animals were mounted onto a stereotaxic device and a median incision was made on the top of the shaved and aseptically prepared skin of head to expose the skull. Two holes were drilled at the parietal region and mounting screws were secured in the holes. An additional hole was drilled at the external occipital crest and used to stereotactically guide the stainless steel cannula into the CM. Dental cement was applied around the cannula and the screws to hold it in place. After light curing and solidification of the cement, the skin wound was sutured with a 4/0 supramid yarn. Correct placement of the cannula is confirmed by spontaneous flow of cerebrospinal fluid (CSF). The rats were removed from the stereotaxic apparatus, received appropriate post-operative care and analgesic treatment and allowed to recover for at least one week until no sign of blood in the CSF was observed. Wistar rats (Crl:WI/Han) were obtained from Charles River (France). All rats were kept under specific pathogen-free conditions. Animals with blood contaminated CSF or in which flow of CSF through the sampling cannula dried out were excluded. Animals were randomized based on similar weight and comparable flow of CSF through the sampling cannula. All animal experiments were approved by the Swiss Veterinary Office Basel-Stadt and were carried out in accordance with the animal permission #2474 (Assessment of active brain transport by measuring the level of therapeutic candidates in the rat CSF and brain).

### Serial collection of CSF and blood

CSF and blood was collected from non-anesthetized rats at different time points as previously described[10]. Blood was collected in EDTA coated tubes (ThermoFisher) and plasma samples were obtained after spinning freshly collected blood at 2000 x g for 10 minutes. CSF was collected in protein low-binding tubes (Eppendorf). Plasma and CSF were then immediately frozen and stored at −80°C until use.

### Pharmacokinetic assays

Construct`s concentrations were assessed by ELISA. Nunc Maxisorp microtiterplates (Sigma) were coated with 500 ng/mL MAb-H-IgG (Biologics Research, Protein Bioanalytics, Roche Penzberg) for 1 h at room temperature on plate shaker at 500 rpm. After washing (wash buffer: PBS / 0.05% Tween 20 Detergent / 0.002% Bronidox reagent) three times, brain, CSF and plasma samples were added to the wells diluted in assay buffer (1 x PBS, 0.5% BSA, 0.002% Bronidox; plasma 1:10000, CSF 1:100, brain homogenate 1:100 for 1 h at room temperature on plate shaker at 250 rpm. The plate was then washed three times and incubated with the detection antibody M-R10Z8E9-IgG- 30ng/ml (Abcam), for 1 h at room temperature on a plate shaker at 250rpm. After a set of washes, the plate was incubated with 5 mU/mL Anti-Digoxigenin-POD (poly), Fab fragments (Second detection reagent) (Roche, 11633716001), and incubated for 1 h at room temperature on a plate shaker at 250rpm. After three wash steps, OX26-NEP was detected by incubation with TMB substrate solution (Roche, 11484281001) for up to 20 minutes. Absorbance was read at 450nm after stopping color development with 1M H2SO4.

### Pharmacodynamic assays

Functionality of the OX26-NEP complex was assessed by Aβ_1–40_ ELISA according to the manufacturer protocol (Wako, 294–64701). Briefly, samples were diluted in standard diluent (CSF, 1:23, plasma, 1:25, brain homogenate 1:50) and incubated overnight at 4 °C on 96-well plates coated with capture antibody BNT77. After the wash steps, HRP-conjugated BA27 antibody was added and incubated for 2 hours at 4 °C, followed by five wash steps. Aβ_1–40_ was detected by incubation in TMB solution for 30 minutes at room temperature. Absorbance was read out at 450 nm after stopping color development with stop solution. Plasma and Brain homogenate samples underwent solid phase extraction before Aβ_1–40_ ELISA. Plasma was added to 0.2% DEA (Sigma) in 96-well plate and incubated for 30 minutes at room temperature. After washing the SPE plate (Oasis, 186000679) with 100% methanol followed by water, plasma samples were added to the SPE plate and any liquid was removed. The samples were washed (5% methanol followed by 30% methanol) and eluted in 2% NH4OH/90% Methanol. After drying the eluates at 55 °C for 99 minutes under constant N2-flow, the samples were reconstituted in standard diluent and Aβ_1–40_ measured as indicated above.

## Results

### Retained enzymatic activity and preserved TfR binding of the BS-NEP fusion protein

**Fig 1:**
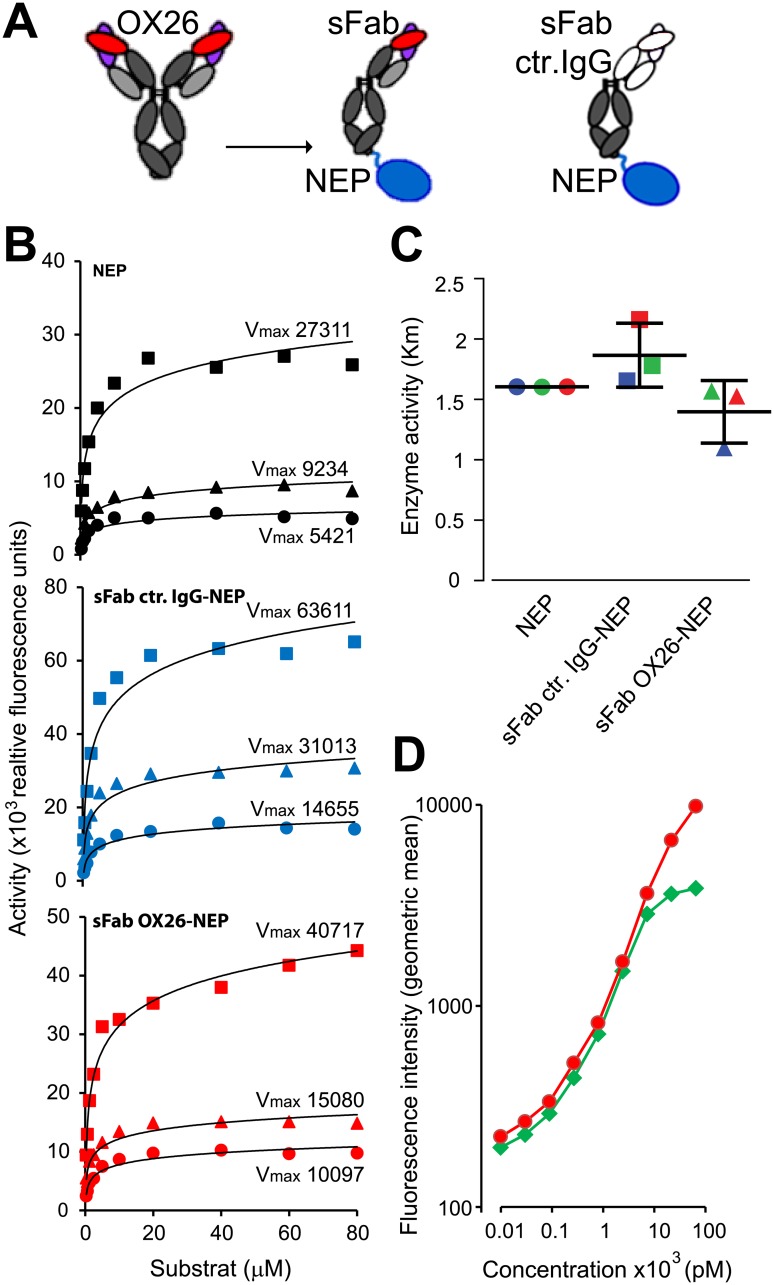
Recombinant sFab OX26-NEP fusion protein is active *in vitro*. (A) Schematic Representation of the OX26 antibody, the one-armed OX26-NEP (sFab OX26) and control IgG-NEP (sFab ctr IgG) fusion constructs. The OX26 Fab fragment (red/magenta) that binds the TfR or the inert control Fab (white) are fused over the C-terminal end of the Fc region (gray) to the human NEP (blue). (B) Enzymatic activity measurements for NEP (black), sFab ctr IgG-NEP (blue) and sFab OX26-NEP (red) at construct concentrations of 4nM (squares), 2nM (triangles) and 1nM (circles). NEP activity constructs were assayed using serial dilutions of a cleavable peptide releasing a fluorescent signal upon cleavage of the substrate. (C) Average *K*_*M*_ values for the three constructs determined based on their averaged EC_50_ values derived from the enzymatic activity measurements at 4nM (green), 2nM (blue) and 1nM (red) for the NEP enzyme (circles), single-armed control IgG-NEP (squares) and single-armed OX26-NEP (triangles). (D) Indirect flow cytometry shows binding to rTfR on C6 rat glioma cells with increasing concentrations of OX26 IgG (green diamonds) and sFab OX26-NEP construct (red circles).

The recombinant fusion proteins were generated by fusing a one–armed OX26 anti-rat TfR antibody (sFab OX26) or a one-armed antibody directed against an irrelevant antigen (sFab ctr IgG) to the extracellular catalytic domain of hNEP (Fig 1A). We included an Fc region to promote long plasma half-life. The maximal reaction velocity (Vmax) (Fig 1B) and the Michaelis-Menten constant (*K*_*M*_) (Fig 1C) were determined *in vitro* by incubating 1nM, 2nM and 4nM of NEP and the sFab IgG NEP fusions with varying concentrations of a fluorogenic NEP reporter substrate (0–160μM). The maximal NEP enzymatic activity for the sFab ctr IgG-NEP was increased in average by 280% and 166% for the sFab OX26-NEP whereas their *K*_*M*_ values were similar compared to the unfused NEP enzyme (1.3 ± 0.3 μM). The retention of the TfR binding affinity for the OX26 IgG and the sFab OX26-NEP construct was measured in a flow cytometric setup using rat TfR expressing rat glioma cells (Fig 1D). Overall, both constructs showed comparable binding to the rat TfR and NEP enzymatic activity.

### CNS delivery of NEP and Aβ_40_ reduction in plasma, CSF and brain parenchyma

**Fig 2:**
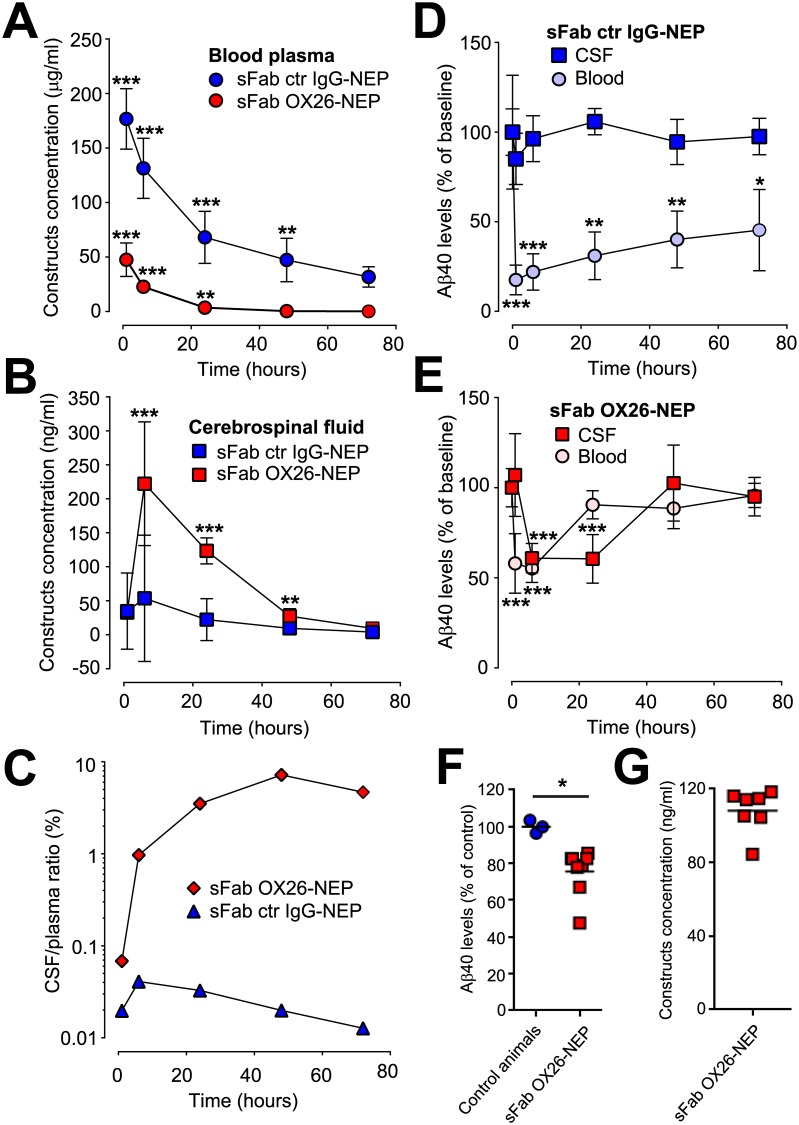
Delivery of NEP to the brain results in CSF Aβ_40_ reduction. Pharmacokinetic, brain uptake and NEP activity of a one-armed OX26 (red) and control IgG (blue) NEP fusion protein in cisterna magna cannulated rats after a 10mg/kg single i.v. injection. Changes in (A) plasma and (B) CSF concentrations of sFab OX26-NEP (red circles and red squares) and sFab ctr IgG-NEP (blue circles & blue squares) following i.v. injection. Shown are average concentrations per treatment group (N = 6) and the SD. Two tailed unpaired t-tests based on the corresponding 72 h concentrations were applied, *** p≤0.001 and ** p≤0.01. Data only presented for CSF samples without blood contamination. (C) The ratio of *in vivo* CSF to plasma concentration ratios of sFab OX26-NEP (red diamonds) and sFab ctr IgG-NEP (blue triangles). (D and E) Aβ_40_ levels in the blood (light colored circles) and CSF (dark colored squares) were measured for (D) sFab ctr IgG-NEP (blue) and (E) sFab OX26-NEP (red) at the indicated time points before and after injection. Shown are percentage levels (±SD) of the groups (N = 6) related to the corresponding 100% baseline Aβ_40_ concentrations before injection. Two tailed unpaired t-tests based on the corresponding 100% baseline concentrations were applied, *** p≤0.001, ** p≤0.01 and * p≤0.05. (F) Percentage Aβ_40_ levels in brain parenchyma of 3 age matched naïve rats (blue circles) and 24 hours after sFab OX26-NEP (red squares) injection (N = 7), and the (G) corresponding brain concentrations of the sFab OX26-NEP construct. Two tailed t-tests based on the corresponding 100% naïve control animals were applied, * p≤0.05.

Cisterna magna (CM) cannulated non-transgenic rats[10] with physiological levels of Aβ were used to determine the pharmacokinetics (PK) and pharmacodynamics (PD) of the two single-armed antibody NEP constructs in the plasma and CSF. Because NEP efficiently cleaves monomeric and oligomeric Aβ_40_[11] we used Aβ_40_ levels as PD marker of NEP activity. First, we measured plasma PK for both one-armed OX26-NEP constructs after a single 10mg/kg i.v. injection (Fig 2A). Clearance of the sFab OX26-NEP was 4 times higher and plasma half-life 3.8 times shorter (t_1/2_ = 6 and 23 h, respectively) compared to the sFab ctr IgG-NEP fusion protein driven by TfR-mediated drug disposition. We observed an increased CSF exposure of the sFab OX26-NEP between 6 h and 48 h after peripheral administration with a 4 times longer CSF half-life (CSF t_1/2_ = 26 h) compared to plasma (Fig 2B). This indicates a more rapid clearance of the sFab OX26-NEP from the plasma compared to the CSF compartment. The low CSF-to-plasma ratio (C_max_ = 0.04% at 6 h) for the sFab ctr IgG-NEP confirms previous reports of low NEP CNS penetration[12]. The CSF-to-plasma ratio for sFab OX26-NEP was much higher (C_max_ = 7.2% at 48 h) compared to sFab ctr IgG-NEP, showing that the one-armed OX26 facilitates the delivery of NEP to the CSF when administered peripherally (Fig 2C). Both, the sFab ctr IgG-NEP (Fig 2D) and sFab OX26-NEP (Fig 2E) led to rapid Aβ_40_ reduction in plasma already 10 minutes after administration, showing that both constructs retained their proteolytic activity *in vivo*. The peripheral Aβ_40_ reduction was observed during the entire observation period for the sFab ctr IgG-NEP. After initial strong plasma Aβ_40_ reduction by sFab ctr IgG-NEP, the Aβ_40_ levels returned to 50% of baseline (24±5 pg/ml Aβ_40_ within 72 h). However, no significant lowering of Aβ_40_ was observed in the CSF for the sFab ctr IgG-NEP construct (Fig 2D). This is in accordance with previous studies in which strong and prolonged NEP mediated peripheral Aβ_40_ clearance did not result into reduced CSF Aβ_40_ levels[12]. The lower plasma exposure of sFab OX26-NEP is reflected in a reduced peripheral Aβ_40_ reduction compared to sFab ctr IgG-NEP. CSF Aβ_40_ levels in animals dosed with sFab OX26-NEP decreased by up to 45% within 24 h and then recovered to pre-dose levels 48 h post dosing. Interestingly, we observed a faster Aβ_40_ reduction in plasma compared to CSF, suggesting that the construct is first exposed and active in the periphery before it enters the CNS compartment (Fig 2E). Furthermore, the Aβ_40_ levels in the CSF compartment are significantly reduced at 24 h whereas the Aβ_40_ levels in the plasma have returned to baseline levels. This could reflect the transport kinetics via the endogenous TfR into the brain. In addition, a significant lowering of Aβ_40_ levels was also observed in the brain parenchyma 24 h after sFab OX26-NEP i.v. injection (Fig 2F). Similar sFab OX26-NEP construct concentrations were measured in the brain parenchyma compared to the CSF 24 h post i.v. injection (Fig 2G). This indicates an uptake and activity of the sFab OX26-NEP fusion protein in the brain parenchyma.

### Different Aβ_40_ reduction kinetics in plasma and CSF

**Fig 3:**
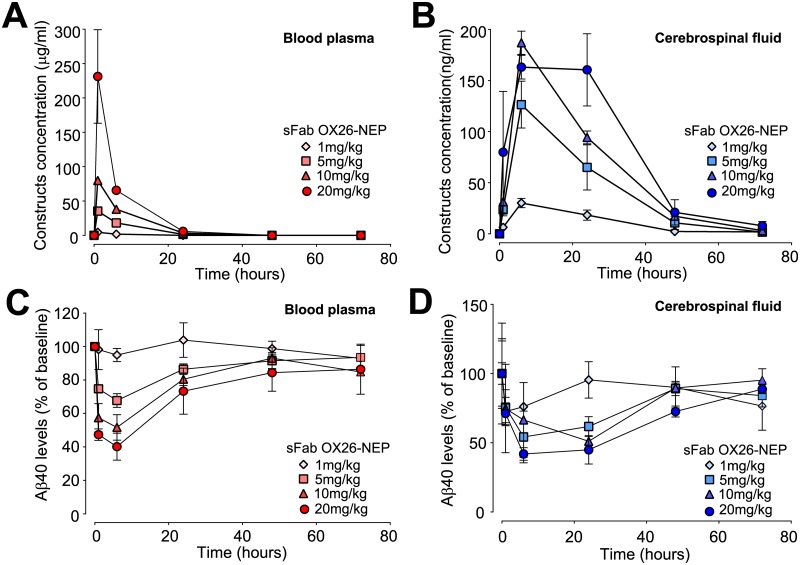
Dose dependent Aβ_40_ reduction in plasma and CSF. Single i.v. administration of 1 mg/kg (diamonds), 5mg/kg (squares), 10mg/kg (triangles) and 20mg/kg (circles) sFab OX26-NEP construct in cisterna magna cannulated rats. (A & B) Construct`s concentrations and (C & D) Aβ_40_ levels measured in blood plasma (red symbols) and CSF (blue symbols) over time.

Next we examined sFab OX26-NEP dose-response in plasma and CSF to determine the minimal effective dose for peripheral and central Aβ_40_ lowering. A single i.v. injection of sFab OX26-NEP was given at doses of 1, 5, 10 and 20mg/kg and construct concentrations were measured in plasma (Fig 3A) and CSF (Fig 3B). A dose dependent clearance of Aβ_40_ was observed in plasma (Fig 3C) and CSF (Fig 3D). The maximal Aβ_40_ reduction in plasma from the pre-dose levels of 117±1pg/ml to 40±1pg/ml and in CSF from 1600±80pg/ml to 980±30pg/ml was observed with the 20 mg/kg dose at 6 h. Hence, Aβ_40_ was not fully depleted in plasma and CSF for the entire duration of the study at the highest dose level. Independent of the dose, the C_max_ (maximum concentration) for the sFab OX26-NEP construct in plasma is around 1 h post i.v. injection (Fig 3A) whereas the C_max_ in CSF is at 6 h (Fig 3B). The constructs at all doses decreased below limit of detection in plasma at 24 h and in CSF at 48 h after injection. Aβ_40_ reduction increased with increasing doses in plasma (Fig 3C) and CSF (Fig 3D). At 24 h the plasma Aβ_40_ levels were still reduced for the three highest doses and returned to pre-dose levels at 48 h. At 48 h the CSF Aβ_40_ levels were still reduced for the highest dose.

### Sustained Aβ_40_ reduction in CSF with multiple dosing

**Fig 4:**
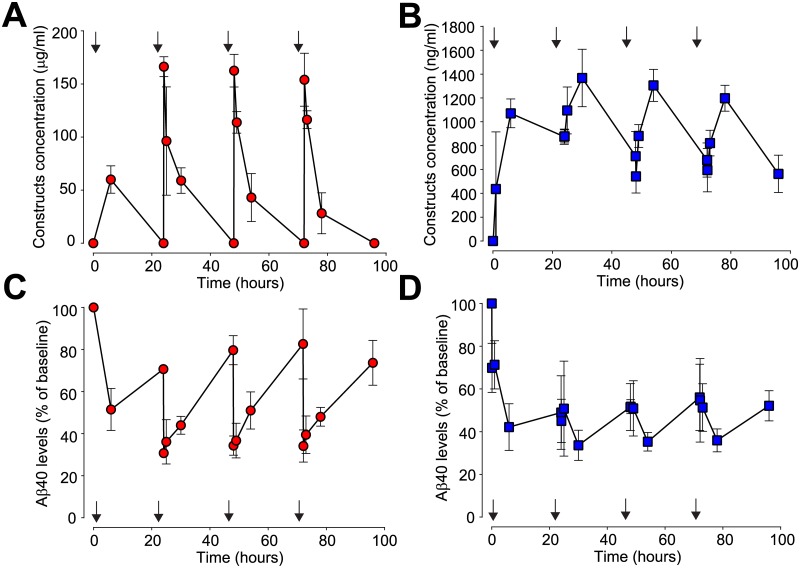
Prolonged Aβ_40_ reduction in plasma and CSF after repeated dosing. (A and B) Pharmacokinetic parameters observed in (A) blood (red circles) and (B) CSF (blue squares) after repeated 10mg/kg i.v. injections (arrows) of sFab OX26-NEP in cisterna magna cannulated rats (N = 3). (C and D) Aβ_40_ levels measured in (C) blood plasma (red circles) and (D) CSF (blue squares).

Following 4 repeated once-daily administrations of 10m/kg sFab OX26-NEP the construct concentration in plasma (Fig 4A) and in CSF (Fig 4B), as well as the Aβ_40_ levels in plasma (Fig 4C) and CSF (Fig 4D) were measured. The mean plasma construct concentration decreased to baseline levels before dosing and the last three dosing intervals led to similar peak plasma construct concentrations (Fig 4A). Despite this, the CSF construct concentrations did not decrease to baseline between the doses (Fig 4B). Peak construct levels measured throughout the study were 6.8nM in CSF and 1.1μM in plasma. In plasma the calculated (AUC based) overall exposure for 96 h was 3.47mg*h/ml and in CSF it was 98.96μg*h/ml. Based on this the CSF/plasma ratios are 1.8% for 6 h, 2.1% for 24 h and 2.9% for 96 h, respectively. 6 h after the first dose, mean plasma Aβ_40_ levels decreased to 50% and each subsequent injections further reduced plasma Aβ_40_ to 30% of baseline levels (12.9pM) (Fig 4C). Initial injection reduced CSF Aβ_40_ to 40% of baseline (644pM) and subsequent injections maintained this reduction with minor fluctuation throughout the study (Fig 4D). Over the entire study duration, we observed a AUC based Aβ_40_ reduction in plasma of 37% and in CSF of 50%.

## Discussion

NEP is an enzyme that breaks down the Aβ peptide[13], which forms amyloid plaques in the brain and has received much attention as a viable target for AD[14]. NEP levels in the human brain decline with aging and in AD could contribute to higher Aβ peptide levels[15]. Animal studies show that genetic modulation of NEP expression and activity in the brain leads to changes in brain Aβ peptide levels[16,17]. However, NEP gene or enzyme systemic delivery to the brain has failed due to the impermeable BBB. Peripheral administration of Fc-hNEP construct in rodents and monkeys led to sustained reduction of Aβ_40_ in blood but without any drop in Aβ_40_ levels in the brain[12]. However, intracranial injection of NEP resulted in an acute decrease in soluble Aβ in the brain. In the present study, we took an advanced approach with a TfR-targeted hNEP. The brain directed construct was assessed in a CM cannulated rat model to study the relationships of Aβ lowering in plasma, CSF and brain parenchyma. We have previously shown that the BS technology allows efficient delivery of antibodies to the brain.[7,18] We propose that this enhanced brain delivery is a direct consequence of the monovalent engagement of the BS construct with the highly expressed TfR on the brain endothelium. On this basis we genetically fused a rat selective OX26 anti-TfR antibody to hNEP. This to investigate if the BS technology also mediates brain deliver of an enzyme. Despite extensive investigations using the OX26 antibody as a BBB-targeting vector, conflicting results have been published regarding the potential of this standard antibody to mediate transport of large molecules into the brain[19]. In order to investigate the impact of a monovalent OX26 construct in a rat model we used the BS technology with NEP as the cargo.

CSF Aβ as a biomarker is regularly used to evaluate PD effects in AD patients. We show that a peripherally administered sFab OX26-NEP construct is effective at degrading Aβ_40_ not only in the blood but also in the CSF in a dose-dependent manner and by repeated dosing. The presence and activity of the sFab OX26-NEP construct in the CSF as well as brain parenchyma supports the concept of facilitated uptake of NEP by a monovalent BS into the brain. Based on other studies utilizing a species-selective mouse anti-TfR antibody (8D3) and the rat selective OX26, it is most likely that the sFab OX26-NEP construct gains access to the CNS by TfR-mediated transport at the BBB. There is some evidence of TfR expression at the choroid plexus[18] and iron transport across the blood-CSF barriers which makes it possible that transport of sFab OX26-NEP through the choroid plexuses may also account for some of the construct-related activity in the CSF. The brain exposure of a TfR BS *in vivo* is not only affected by efficiency of transcytosis across the BBB but also by its plasma half-life. Despite the fast plasma clearance of the sFab OX26-NEP construct we observed effective delivery of the enzymatically active NEP payload into the brain. The short plasma half-life of the sFab OX26-NEP construct is either due to target-mediated drug deposition (TMDD) through TfR on various peripheral cells or a clearance through the glycol-profile on NEP. It was previously shown that a reduction in affinity of a bivalent OX26 was sufficient to prolong the plasma half-life and improve the brain exposure[20]. The longer plasma half-life is likely explaining at least in part the improved brain expose as it provides TfR binders over an extended time period circulating in the peripheral compartment. Thus, reduced affinity of a monovalent OX26 could therefore result in a more favorable PK profile and increased and prolonged accumulation in the brain.

Our results show that the exposure of sFab OX26-NEP in the CSF is delayed compared to the blood compartment. This is probably due to its transport across the BBB and the drainage along the interstitial fluid into the CSF. One must bear in mind that some neuropils also express a low level of TfR[21]. Accordingly, fusion proteins like sFab OX26-NEP might in fact target both neuronal Aβ_40_ as well as neuronal TfR and may become unavailable for drainage into the CSF. Additional studies are required to map sFab OX26-NEP in the brain parenchyma after administration. We observed a partial depletion of soluble Aβ_40_ in the CSF and a slightly stronger effect in the blood compartment, with a robust return to baseline following sFab OX26-NEP injection. The incomplete Aβ_40_ removal might be due to either rapid Aβ re-synthesis in the brain, Aβ might be in a compartment inaccessible for NEP and/or the rapid clearance of NEP in both plasma and CSF. These factors need to be taken into consideration, since it will affect PK/PD relationship of NEP. In our non-transgenic rat model, we neither monitored the levels of multimeric Aβ species nor proteolytic Aβ_40_ fragments. It would be important to investigate whether NEP mediated reduction of soluble Aβ_40_ has an effect on Aβ oligomer concentration in the brain of AD animal models. Overall our data suggest that by taking a brain targeted approach using the BS technology it is possible to achieve sufficient NEP concentrations in the brain to strongly reduce Aβ_40_ levels in both CSF and brain parenchyma.

A major challenge for treatment of many brain diseases is to overcome the impediment of delivery of therapeutic macromolecules to the brain. Here, we show that by applying the Brain Shuttle technology, we successfully display effective retained enzymatic activity and transport of NEP across the BBB into the CNS. Systemic administration of BS-NEP not only rapidly and strongly lowers Aβ in the periphery but also in the CNS where the pathology exists and progresses. This approach constitutes a unique avenue, not only for Alzheimer`s disease but for a broad spectrum of neurological diseases.
